# The significance of reduction of valgus-intercalated femoral neck fracture with valgus angle > 15°and the selection of internal fixation by finite element analysis

**DOI:** 10.1186/s12891-024-07180-z

**Published:** 2024-01-20

**Authors:** Alimujiang Yusufu, Tusongjiang Yusupu, Abuduwupuer Haibier, Abudula Abulaiti, Jian Ran

**Affiliations:** 1 Department of OrthopedICS of Trauma, Sixth Affiliated Hospital of Xinjiang Medical University, Orthopaedic Hospital of Xinjiang Uygur Autonomous Region, No.39 Wuxing Road, Urumqi, People’s Republic of China; 2 Xinjiang Medical University, Urumqi, Xinjiang Uygur Autonomous Region People’s Republic of China

**Keywords:** Femoral neck fracture, Valgus, Insertion, Finite element analysis

## Abstract

**Background:**

Currently, consensus is lacking on the necessity of internal fixation after reducing valgus-intercalated femoral neck fractures with abduction > 15°. This study employs finite element analysis to compare the biomechanical differences between the femoral neck dynamic cross nail system (FNS) and inverted cannulated screw (ICS), aiming to provide a foundation for clinical procedures.

**Methods:**

Human femur CT scan data were processed using MimICS21.0 and Geomagic 2021 software, imported into Solidworks2021 to create fracture models, based on Garden I abduction and Valgus-intercalated femoral neck fractures. The internal fixation model was divided into two groups: A—Anatomic reduction group; B—Valgus-intercalated femoral neck fracture group. ANSYS software facilitated meshing, material assignment, and data calculation for stress and displacement comparisons when ICS and FNS were applied in reduction or non-reduction scenarios.

**Results:**

Without internal fixation, peak femur stress in both groups was 142.93 MPa and 183.62 MPa. Post FNS fixation, peak stress was 254.11 MPa and 424.81 MPa; peak stresses for the two FNS models were 141.26 MPa and 248.33 MPa. Maximum displacements for the two FNS groups were 1.91 mm and 1.26 mm, with peak fracture-end stress at 50.751 MPa and 124.47 MPa. After ICS fixation, femur peak stress was 204.76 MPa and 274.08 MPa; maximum displacements were 1.53 mm and 1.15 mm. ICS peak stress was 123.88 MPa and 174.61 MPa; maximum displacements were 1.17 mm and 1.09 mm, with peak fracture-end stress at 61.732 MPa and 104.02 MPa, respectively.

**Conclusions:**

Our finite element study indicates superior mechanical stability with internal fixation after reducing valgus-intercalated femoral neck fractures (> 15°) compared to in situ fixation. Additionally, ICS biomechanical properties are more suitable for this fracture type than FNS.

## Introduction

With the aging population and increasing traffic accidents, the incidence of hip fractures is steadily rising. Epidemiological projections estimate the occurrence of 4 million hip fractures by 2025, escalating to 6.3 million by 2050 [1]. Approximately 50% of these fractures involve the femoral neck [1–3], of which valgus femoral neck fractures constituting 15% to 29% of these cases [4].

The initial stage of a valgus-intercalated femoral neck fracture typically presents as a Garden I stable fracture. Examination of CT scan and X-ray data reveals it as a complete femoral neck fracture with substantial spatial displacement of the femoral head [5, 6], challenging the conventional notion that an abducted embedded femoral neck fracture is stable and non-displaced. Various treatment modalities exist, ranging from non-operative approaches and in situ internal fixation to closed/open reduction, internal fixation, and joint replacement. However, there is no consensus on the optimal treatment approach. In situ internal fixation offers advantages such as minimally invasive treatment, short operation time, and reduced need for blood transfusion, allowing early-stage functional exercise [7, 8]. Nonetheless, complications like femoral neck shortening and impact on the acetabular lip can diminish clinical efficacy [9, 10]. Some advocate for anatomical reduction to mitigate long-term complications' risks [11]. When the valgus angle exceeds 15° in femoral neck valgus fractures, the entire femoral shaft shortens by 10% [12], increasing the likelihood of stress concentration on internal fixation, ultimately leading to failure and imposing economic and psychological burdens on patients. This study reveals that a greater abduction angle results in a shorter femoral neck, poorer functional recovery, and an elevated risk of osteonecrosis and femoral head reoperation [13]. Therefore, the preoperative valgus angle significantly influences the surgical outcomes.

The cannulated screw (CS) stands out as the primary choice for treating young femoral neck fractures [14]. It can be categorized into positive triangle and inverted triangle structures based on screw arrangement. Some studies suggest that the inverted cannulated screw (ICS) exhibits superior biomechanical stability [15]. The femoral neck dynamic cross nail system (FNS), a recently developed internal fixation system, emerges as an effective alternative for managing unstable femoral neck fractures, surpassing hollow screws [16]. However, a recent review article assessing the efficacy of ICS, FNS, dynamic hip screw (DHS), and various other implants in femoral neck fracture treatment found that each method has its merits and drawbacks, making it challenging to establish superiority [17]. The advantages and disadvantages of ICS and FNS in valgus-intercalated abduction and valgus femoral neck fractures have yet to reach a consensus.

Remarkably, few studies have explored the impact of the valgus angle and internal fixator on the efficacy of femoral neck fracture treatment. Hence, we conducted a comparative analysis to evaluate the significance of reducing valgus angles exceeding 15° and the biomechanical stability of different internal fixators using the finite element method.

## Materials and methods

A healthy adult male, 25 years old, with a height of 172 cm, body mass of 70 kg, and no history of medical, surgical diseases, limb disability, or trauma, was selected for the study. Full-length scans of both lower limbs were conducted using GE64 spiral CT, with each slice having a thickness of 0.6 mm, generating two-dimensional cross-sectional images saved in Dicom format. The research protocol adhered to the Helsinki Declaration and ethical guidelines of the Sixth Affiliated Hospital of Xinjiang Medical University, with ethical approval number LFYLLSC20231013-01. Subjects participated voluntarily, provided informed consent for data collection, and signed the informed consent form.

### Establishment of three-dimensional model of femur.

**Fig. 1:**
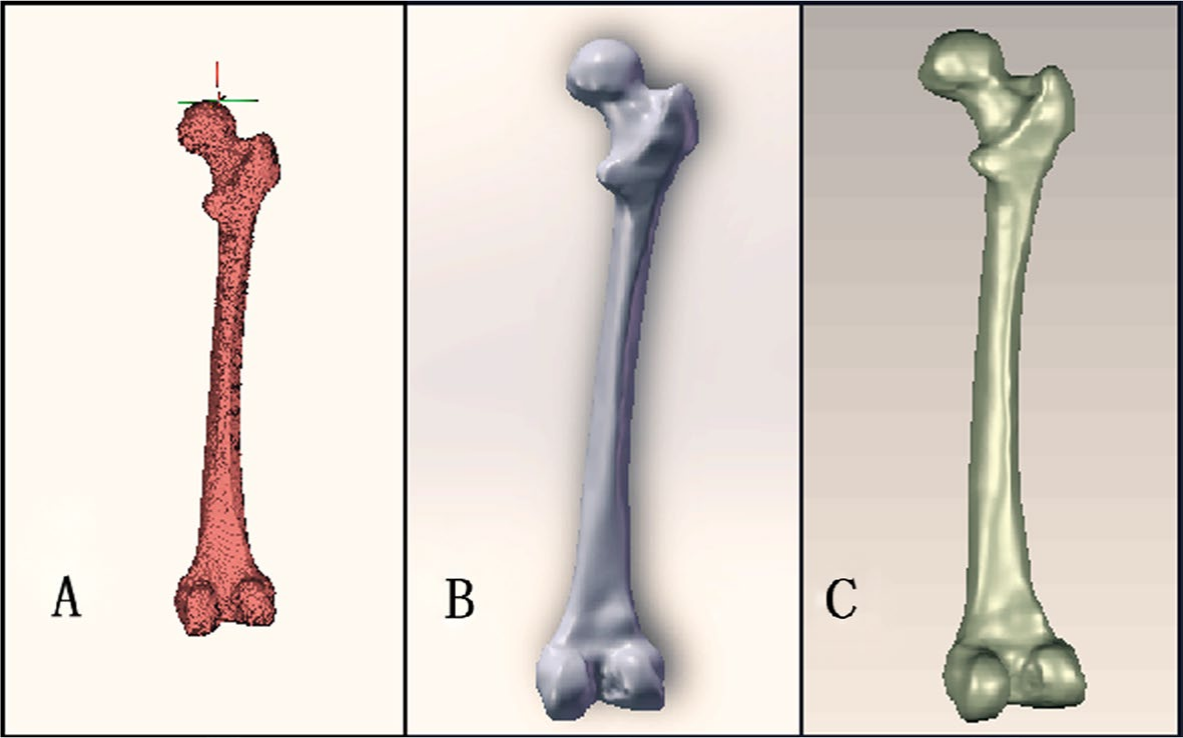
Three-dimensional geometrical modeling; Three-dimensional surface model; 3D entity mode

The Dicom-formatted CT data were imported into MimICS21.0 software. Using the 3Dbone tool, the femur, pelvis, patella, tibia, and fibula were extracted, and other bones were hidden. The femur was preserved, and a three-dimensional model was reconstructed through masking, wrapping, smoothing, and other steps. The model was then exported as an "STL" file. GeomagICStudio was used for further optimization processing of the three-dimensional femur model. This involved steps such as smoothing, nail deletion, frosting, and gridding to assess the non-defective surface. The mesh was redrawn, ensuring accurate curved surfaces, automatic surfacing, and fitting to obtain the femoral cortex. A copy was made to form another model, transformed into polygons, and inwardly biased by 3.64 mm [18]. The femoral cancellous three-dimensional model was obtained through these steps (Fig. 1).

### Establishment of a model of femoral neck fracture

**Fig. 2:**
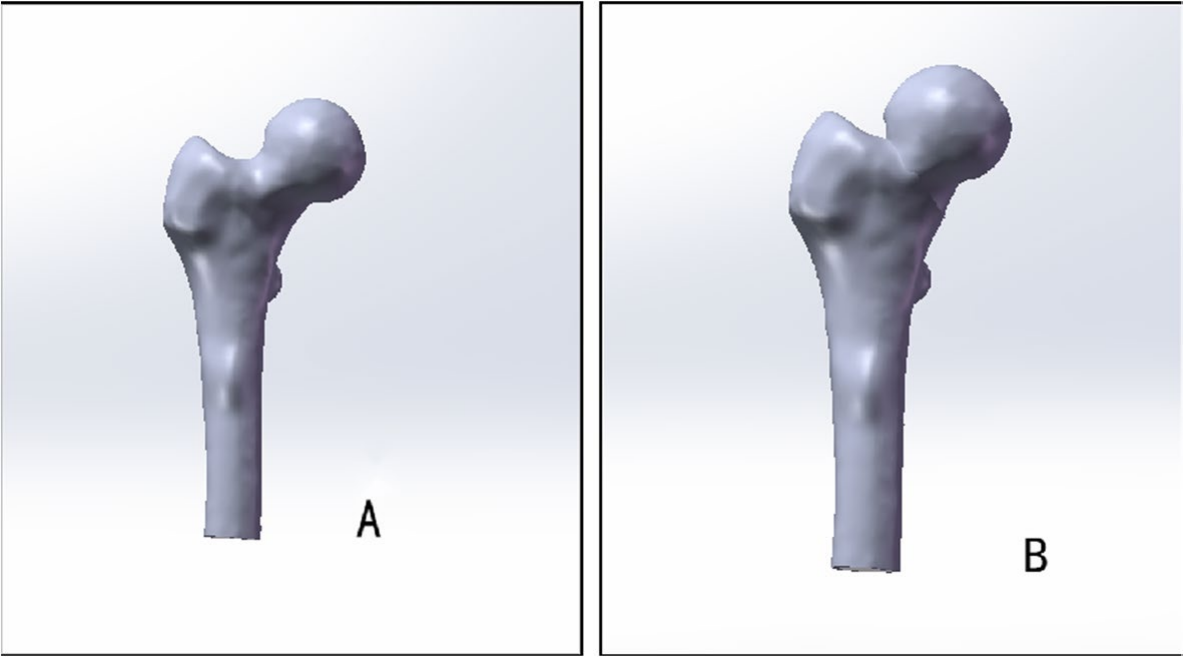
**A** Anatomical reduction of the femoral model; **B** Valgus intercalation model

The aforementioned models were imported into Solidworks (French DassaultSystemes) software for entity reconstruction. Commencing from a specific point on the femoral neck, a parallel datum was established, and the Garden I type of femoral neck fracture (abduction insertion) served as a template to create a straight line intersecting the femoral shaft. Utilizing the segmentation command, the femur was divided into four components: femoral head cortex/cancellous and femoral shaft cortex/cancellous. To streamline calculations, the distal femur was removed, ensuring consistency between the two models. The anatomical reduction group followed the aforementioned process. However, for the valgus intercalation group, a 20° reverse rotation along the Y-axis was applied to the segmented femoral neck and femoral head to establish a model representing valgus > 15° (Garden Index of 180°). Simulation of the acceptable reduction limit was conducted, with a valgus angle of 20° (Garden index 180°) and a posterior inclination angle of 20° (Garden index 160°). To mimic the actual fracture, compression of the cancellous bone in the overlapping region of the femoral head and neck was implemented, as illustrated in Fig. 2A, B.

### Establishment and categorization of Internal Fixation Model.

**Fig. 3:**
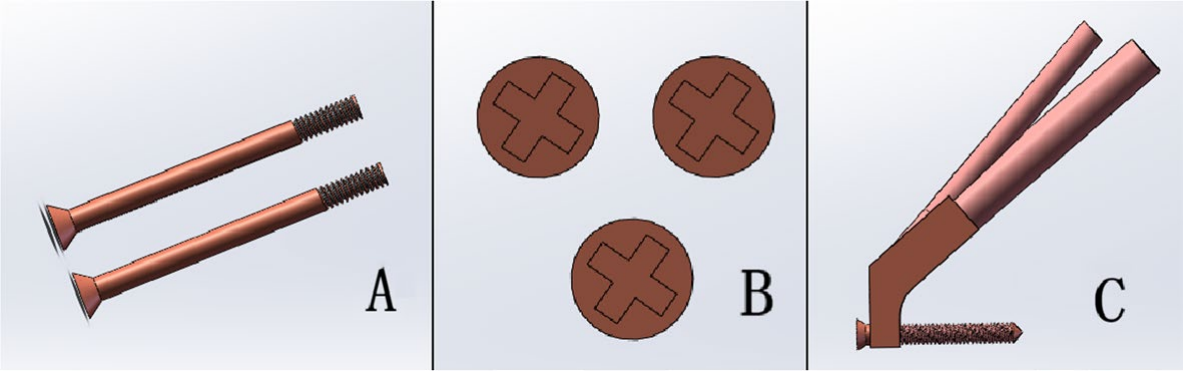
**A** ICS (anterior view); **B** ICS(norma lateralis); **C **FNS(anterior view)

Utilizing Solidworks software for construction based on clinical data, the inverted triangular screw model was established with a screw diameter of 7.3 mm and a thread length of 16 mm. The femoral neck system model, composed of a power rod (diameter 10 mm), anti-rotation screw (diameter 6.5 mm, length 89 mm, positioned at a 7.5° angle to the power rod), locking plate, locking screw, and other components, was assembled (all constructed from titanium alloy). Given that the experiment's focus did not involve the thread, the thread cross-section was simplified to a cylinder. At the distal end, a 5 mm locking screw was employed for fixation (Fig. 3A, B, C). Subsequently, the internal fixation model was integrated into the femoral model in SolidWorks, positioning the power rods under the femoral neck by 5 mm. Boolean operations were applied to remove the screw and bone at the power rod. The aforementioned solid model was automatically meshed in the Ansys module (ANSYS Company, USA), selecting a tetrahedral solid 185 element grid for the mesh.

### The assignment of material properties.

**Fig. 4:**
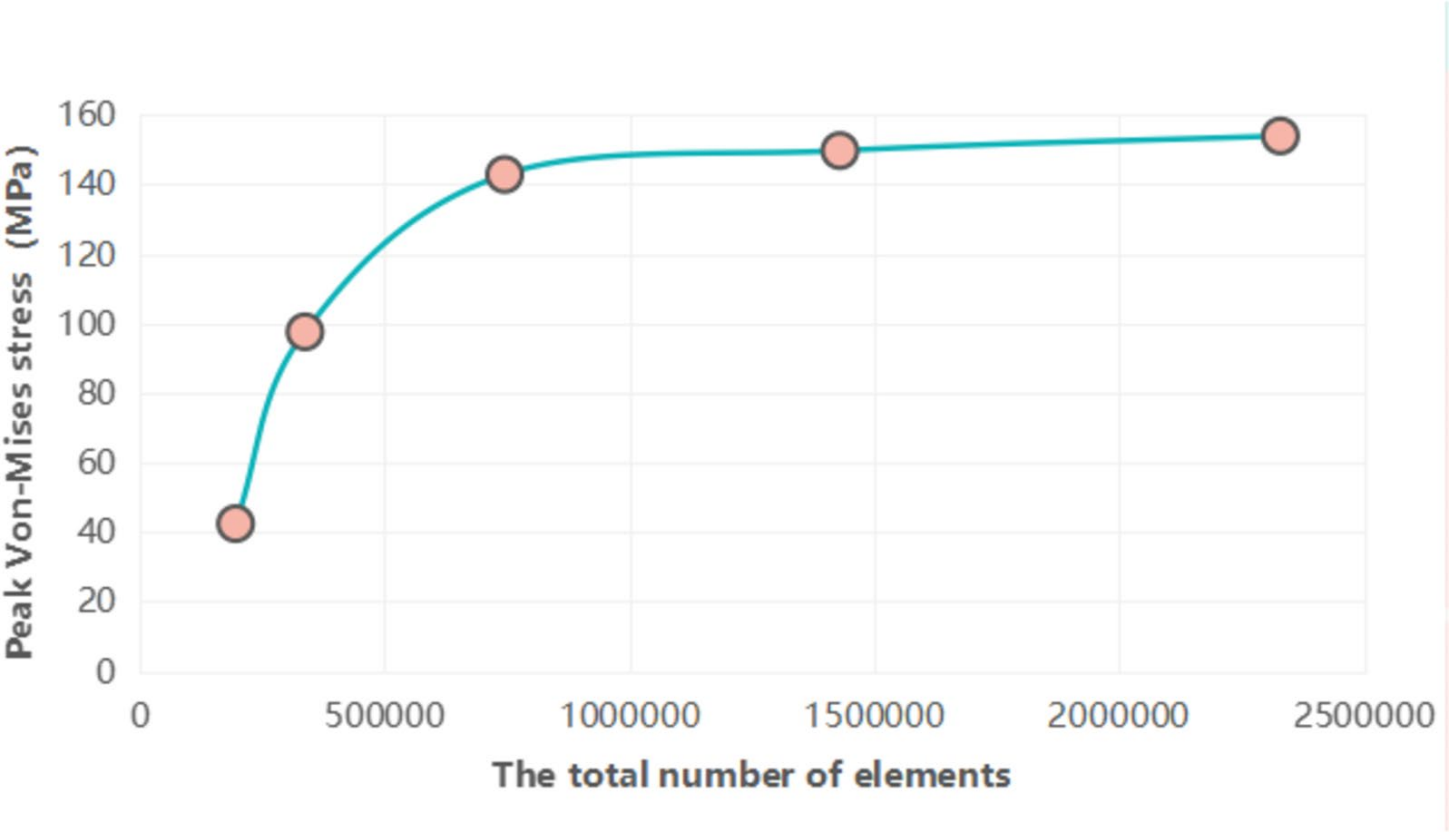
Convergence tests

We generated a tetrahedral overall grid model with a maximum grid size of 2 mm, an internal fixation area in contact with the femur of 1.5 mm, and a final set of 0.5 mm grids. A discrete element model for convergence testing was subsequently executed until the calculated stress deviation was < 5%, as depicted in Fig. 4 This model comprised approximately 734,510 nodes and 2220,835 elements. Assuming all bones and fixers are linear elastic materials with continuity, full elasticity, uniformity, and isotropy, we assigned material properties to the cortical bone, cancellous bone, and internal fixation system of the femur, in line with the literature's specifications [19–23]. The number of nodes and elements in the four models, along with the elastic modulus of the bones and implants, are detailed in Table 1 and 2.

**Table 1:** Material parameters

**Materials**	**Modulus of elasticity (MPA)**	**Poisson's ratio**
Cortical bone	16,800	0.3
Cancellous bone	840	0.2
Internal fixation (titanium alloy)	110,000	0.3

**Table 2:** Number of elements and nodes of finite element model

**Model type**	**ICS**	**FNS**
Anatomic reduction group		
Node	740,687	745,131
Unit	492,217	498,171
Mesh size	Maximum: 2.0 mm; Minimum: 1.5 mm	
valgus-intercalated group		
Node	731,874	737,759
Unit	492,007	492,026
Mesh size	Maximum: 2.0 mm; Minimum: 1.5 mm	

### Boundary and loading conditions.

The contact between the internal fixation and bone, as well as internal fixation itself, was constrained, and friction contact was applied to the fracture surface of the anatomical reduction group, with a friction coefficient set to 0.46 [23]. (The abduction insertion group was characterized by stable contact, as the broken ends of the fracture were intercalated, forming binding contact.) The femur's base was fixed, and degrees of freedom in the X, Y, and Z directions were constrained to prevent rigid body movement.

**Fig. 5:**
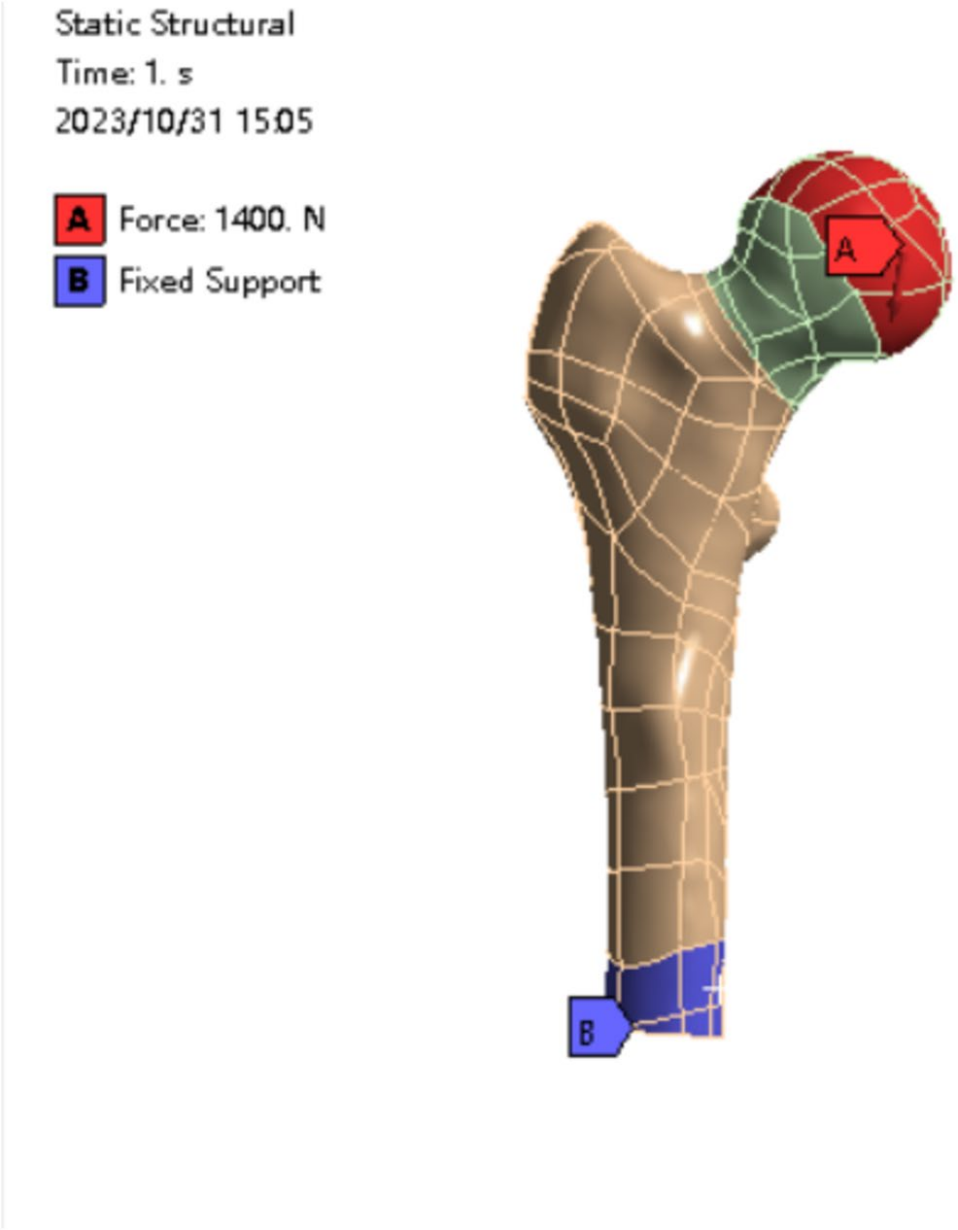
The red grid is the weight center of the femoral head, the red arrow indicates the direction of the femoral force; the blue area is the support site

To authentically replicate the femur's force pattern in daily life, a coordinate system was established in the weight-bearing center region of the femoral head [24]. The x-axis of the coordinate system aligns with the femoral coronal plane at 13° and the sagittal plane at 8°. A load of 1400N was applied along the X-axis [25], simulating the weight-bearing capacity during adult single-leg bearing while walking. Notably, this experimental study did not delve into the specific roles of individual muscle groups. All nodes below the condyle of the distal femur were fully constrained, and distal femoral displacement along the X, Y, and Z axes was set to 0 (Fig. 5). The average mesh size of the constructed femoral model is 2 mm, with refinement around the interface between the femur and internal fixation, ensuring a minimum size above 1.5 mm.

### Main outcome measures.

The simulation calculations were conducted using Ansys 21.0 software, with a primary focus on observing the following aspects: 1. Stress distribution and peak value in femur and internal fixation; 2. Displacement distribution and peak value of femur and internal fixation; 3. Stress distribution and peak value of fracture section.

## Results

### Stress distribution of each model.

#### Von-Mises stress distribution of femur in anatomical reduction group and valgus intercalation group.

**Fig. 6:**
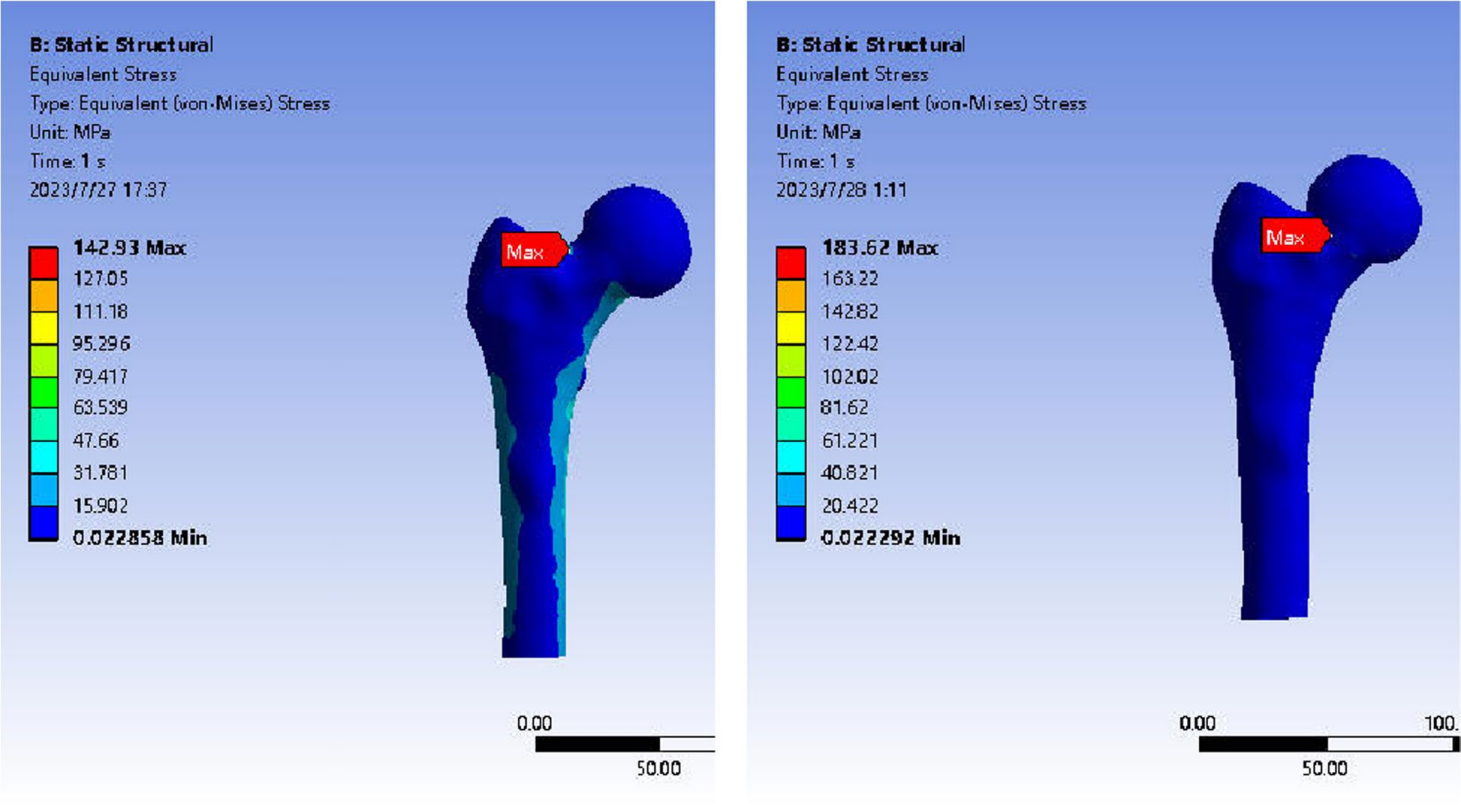
Stress distribution and peak values in the femur were analyzed for cases of anatomical reduction and abduction-embedded without fixation. Note: The left is the anatomical reduction group; the right is the valgus intercalation group

The stress distribution in the models of anatomical reduction and abduction insertion of the femur was assessed without the presence of internal fixation in both groups. In both the reduction group and the in situ group, the maximum stress was observed on the upper lateral side of the fracture end of the femoral neck. Stress concentration occurred near the end of the fracture line and the medial side of the femur, with an even distribution along the fracture line. The peak stress in the reduction group was 142.93 MPa, while in the in situ group, it was 183.62 MPa, indicating a lower peak stress in the reduction group compared to the in situ group (Fig. 6).

#### Von-Mises stress distribution of femur fixed by ICS in anatomical reduction group and valgus intercalation group.

**Fig. 7:**
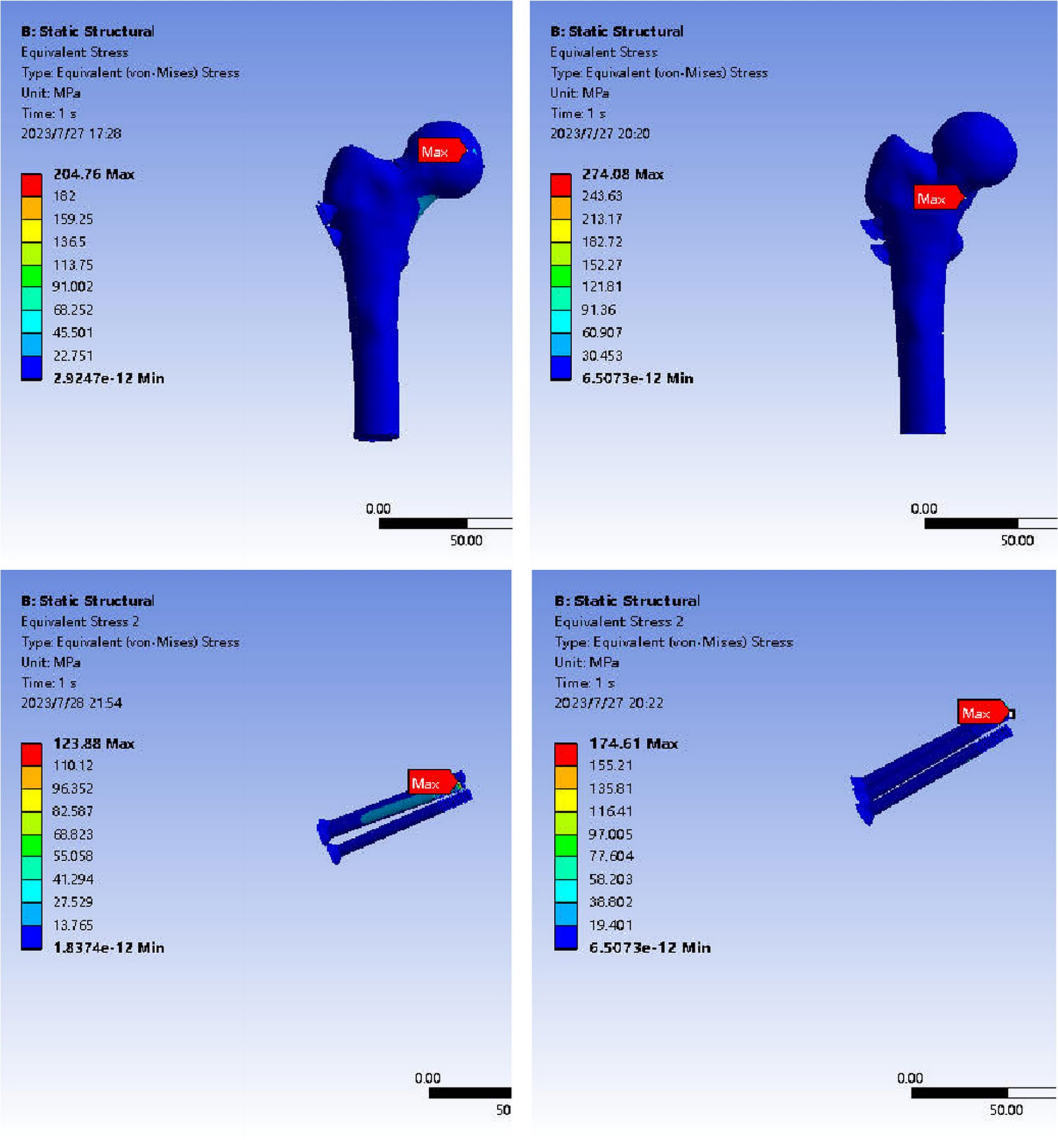
Stress distribution and peak value of anatomical reduction and abduction embedded femur during ICS fixation. Note: The left is the anatomical reduction group; the right is the valgus intercalation group

The stress cloud map analysis following ICS fixation revealed that in the ICS-fixed model, the maximum stress in the femur for the reduction group was primarily distributed in the concave part of the femoral head, reaching a peak stress of 204.76 MPa. In the in situ group, the maximum stress was predominantly distributed in the lower end and medial part of the femoral neck, with a peak stress of 274.08 MPa. The stress distribution in the ICS internal fixation was concentrated on the middle surface of the screw near the fracture line, exhibiting uniform distribution along the screw. The peak stress in the reduction group was 123.88 MPa, whereas in the in situ group, it was 174.61 MPa, indicating a lower peak stress in the reduction group compared to the in situ group (Fig. 7).

#### Von-Mises stress distribution of femur fixed by FNS in anatomical reduction group and valgus intercalation group.

**Fig. 8:**
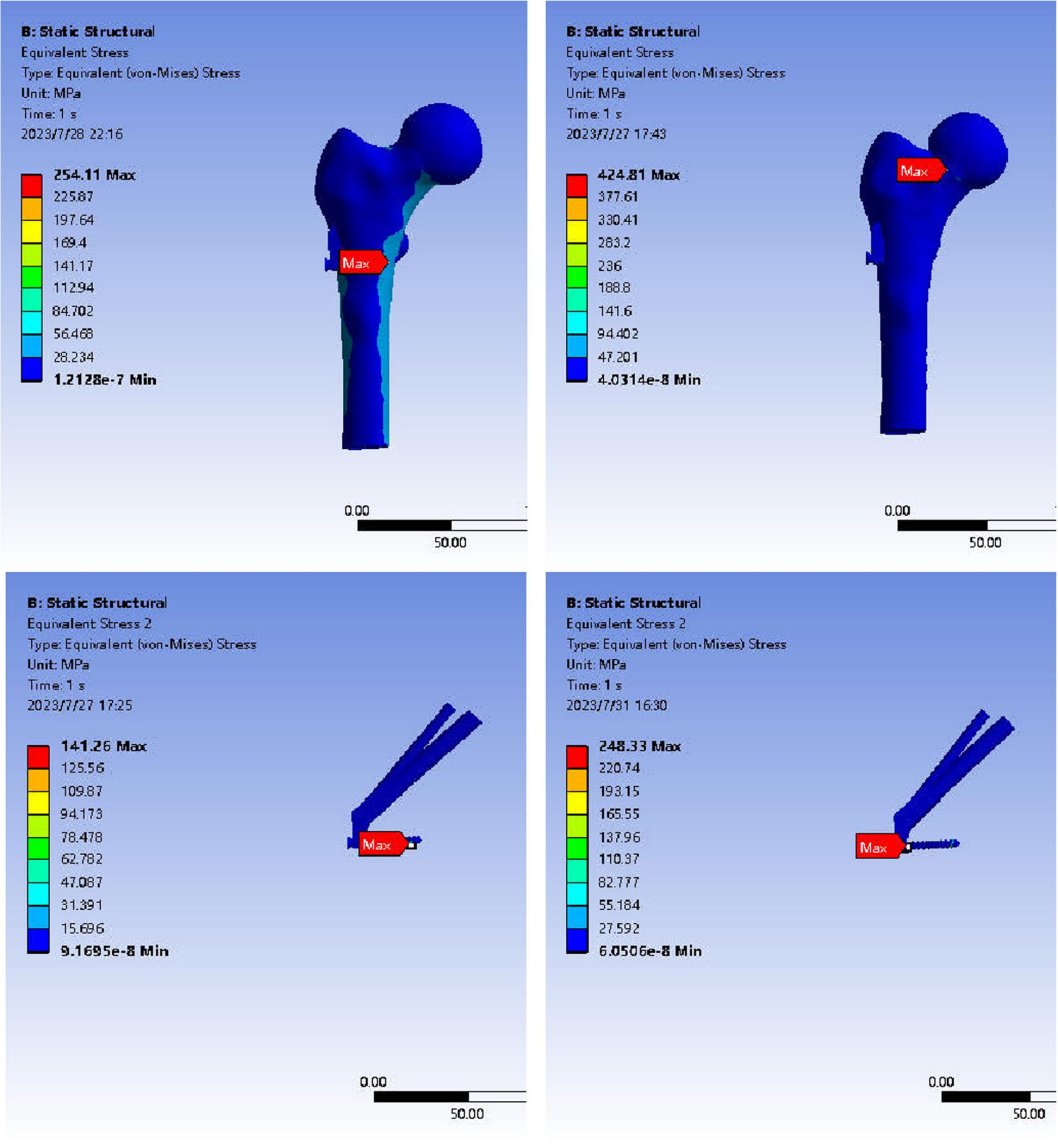
Stress distribution and peak value of anatomical reduction and abduction embedded femur during FNS fixation. Note: The left is the anatomical reduction group; the right is the valgus-intercalated group

The stress cloud map analysis following FNS fixation revealed that in the FNS-fixed model, the maximum stress in the femur for the reduction group was primarily concentrated in the lesser trochanter of the medial femur, peaking at 254.11 MPa. In the valgus intercalation group, the maximum stress was predominantly distributed along the fracture line of the femoral neck, reaching a peak stress of 424.81 MPa. The stress in the FNS internal fixation was centered on the distal screw, with uniform distribution across the internal fixation system. The peak stress in the reduction group was 141.26 MPa, whereas in the valgus intercalation group, it was 248.33 MPa, indicating a lower peak stress in the reduction group compared to the valgus intercalation group (Fig. 8).

#### Von-Mises stress distribution of fracture end in anatomical reduction group and valgus intercalation group.

**Fig. 9:**
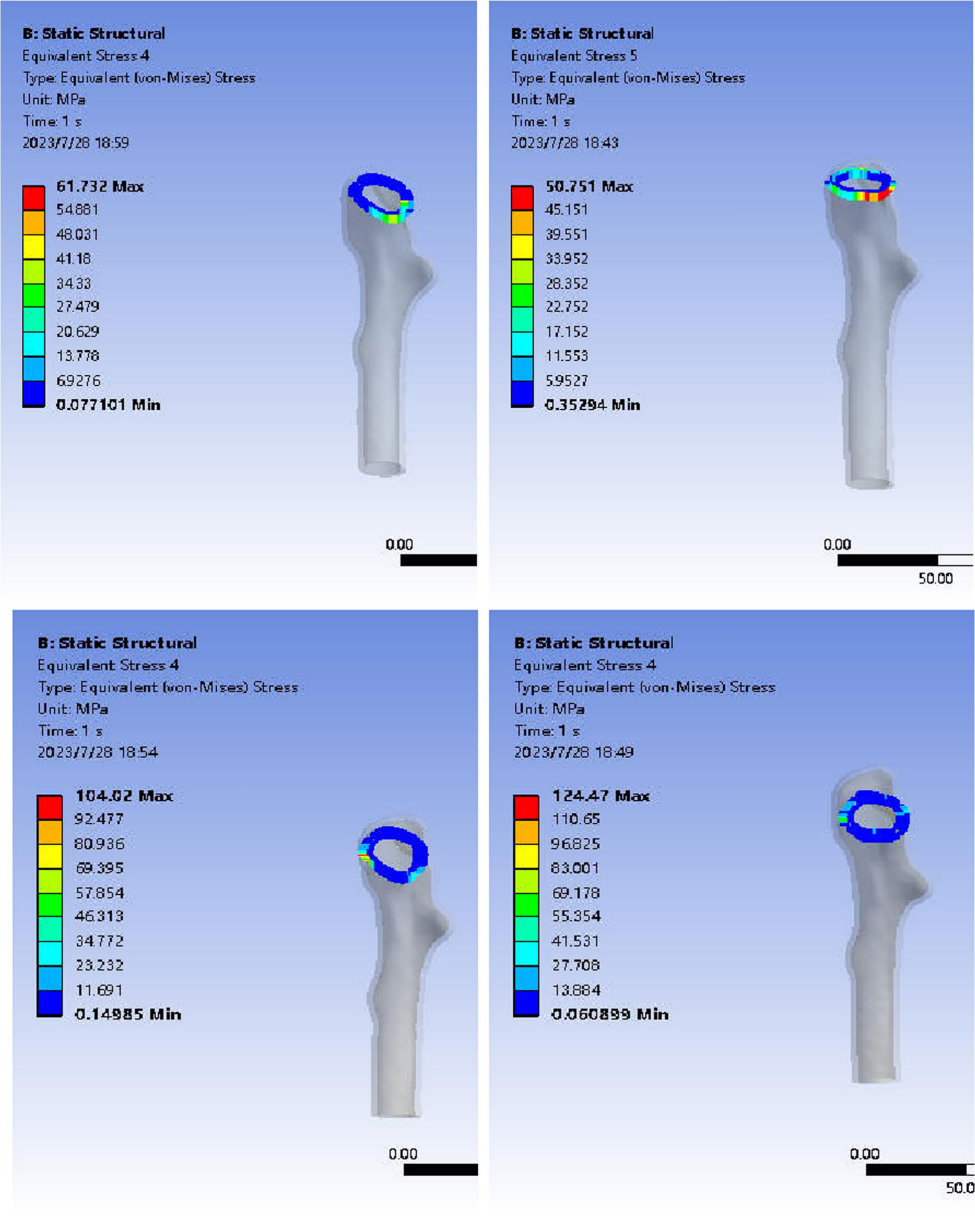
Stress distribution in the femur during ICS and FNS fixation for anatomical reduction and abduction-embedded conditions. Note: The upper left picture depicts the anatomical reduction of the femur fixed by ICS, the lower left picture shows the anatomical reduction of the femur fixed by ICS, the upper right picture illustrates the anatomical reduction of the femur fixed by FNS, and the lower right picture displays the abduction and embedded femur fixed by FNS in the anatomical reduction group

The stress cloud image analysis of the fracture end indicated that the maximum stress in the femoral head of both model groups after ICS fixation was predominantly situated at the junction of the cross-section and the internal fixation. The peak stress in the reduction group was 61.73 MPa, and in the in situ group, it was 104.02 MPa, with the latter exhibiting higher peak stress than the former. Following FNS fixation, the maximum stress in the femoral head of both groups was primarily located on the medial side of the fracture end. The peak stress in the reduction group was 50.75 MPa, and in the valgus intercalation group, it was 124.47 MPa, with the latter showing higher peak stress than the former (Fig. 9).

#### Von-Mises stress peak distribution of FNS and ICS.

**Fig. 10:**
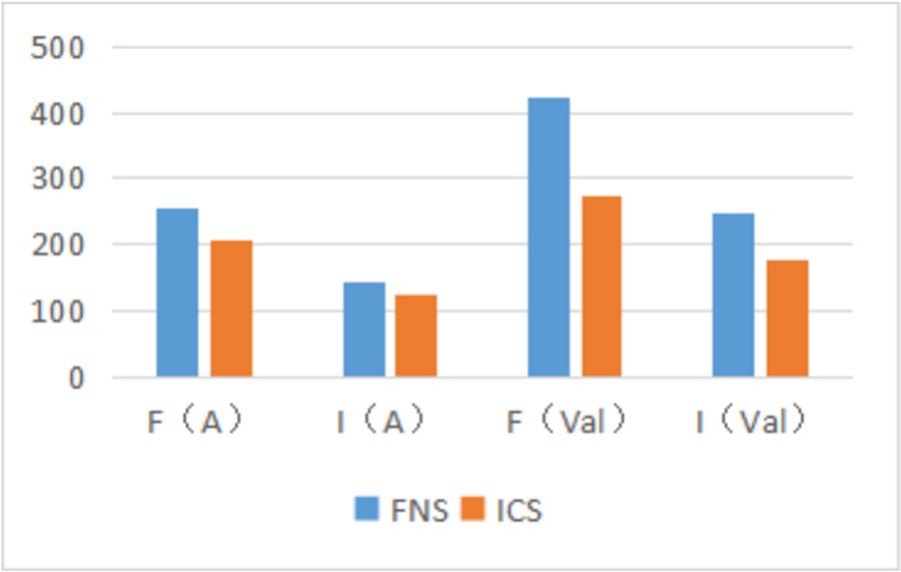
F: femoral model; I: Internal fixture; A: Anatomic reduction group; Val: Valgus intercalation group

The authors conducted a comparison of stress distribution in the femur and internal fixation between the reduction group and valgus intercalation group. In the ICS group, the peak stress (204.76) was significantly lower than that in the FNS group (254.11), and in the ICS group (123.88) of the reduction group, it was significantly lower than the FNS group (141.26). In the valgus intercalation group, the peak stress in the ICS group (274.08) was significantly lower than that in the FNS group (424.81), and the peak stress in the ICS group (174.61) was significantly lower than that in the FNS group (248.33). These findings suggest that ICS exhibits superior stress resistance compared to FNS (Fig. 10).

### Displacement distribution of each model.

#### Displacement distribution of femur and internal fixation in anatomical reduction group and valgus intercalation group after ICS fixation.

**Fig. 11:**
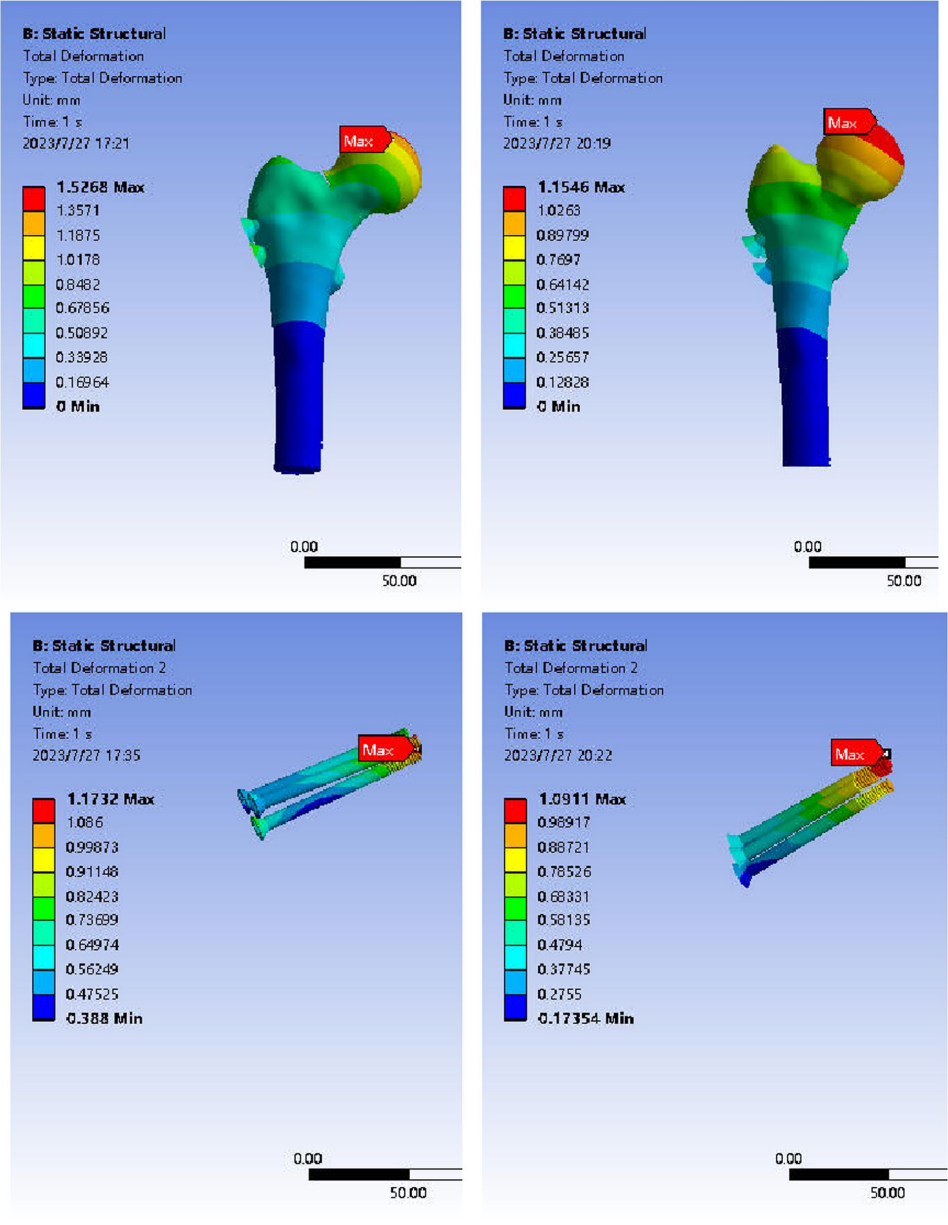
Displacement distribution of anatomical reduction and abduction embedded femur during ICS fixation. Note: The left is the anatomical reduction group; the right is the valgus intercalation group

In the case of abduction and intercalated femoral neck fracture fixed by ICS, the maximum displacement in both the reduction and in situ groups was observed in the upper part of the femoral head. The peak displacement in the two groups was 1.53 mm and 1.15 mm, with the in situ group exhibiting less displacement than the reduction group. Under a 1400N load, the maximum displacement of ICS internal fixation was primarily concentrated at the top of the screw, gradually decreasing in a concentric circle from the head to the tail of the screw. The peak displacement of internal fixation in the reduction and in situ groups was 1.17 mm and 1.09 mm, respectively, with the in situ group showing smaller displacement than the reduction group. Due to the intercalation of the fracture ends in abduction intercalated femoral neck fracture, it demonstrated greater stability than the reduction group. This suggests a lower risk of internal fixation displacement, indirectly reflecting a reduced risk of internal fixation failure to some extent (Fig. 11).

#### Displacement distribution of femur and internal fixation in anatomical reduction group and valgus intercalation group after FNS fixation.

**Fig. 12:**
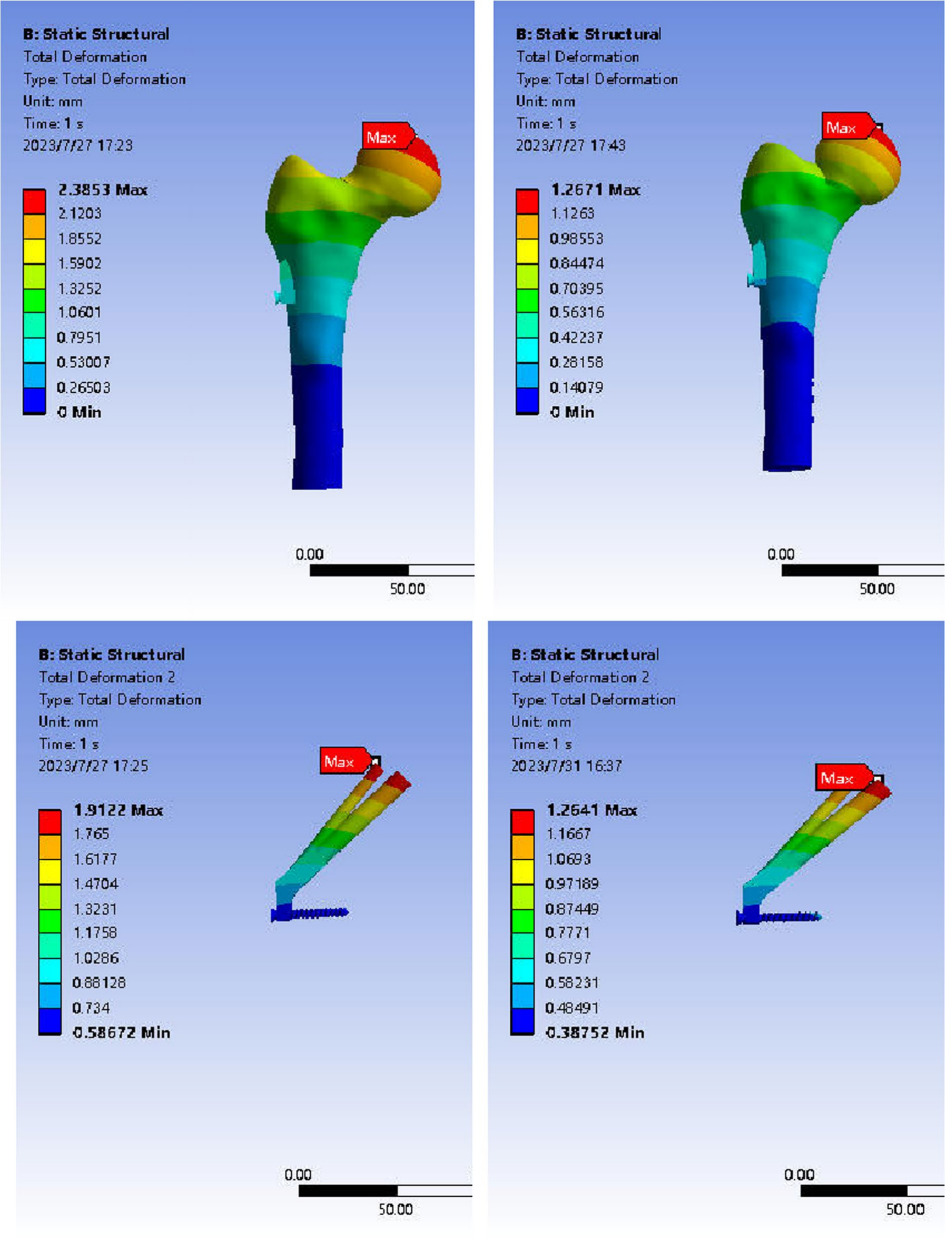
Displacement distribution of anatomical reduction and abduction embedded femur during FNS fixation. Note: The left is the anatomical reduction group; the right is the valgus intercalation group

The results of displacement cloud image analysis revealed that after FNS fixation, the maximum displacement of the femur in both the reduction and in situ groups was concentrated at the top of the femoral head and decreased in a downward direction. The peak displacement of the femur in the two groups was 2.39 mm and 1.27 mm, respectively, with the in situ group showing less displacement than the reduction group. Under the influence of a 1400N load, the maximum displacement of FNS internal fixation was primarily concentrated at the top screw, gradually decreasing from the top to the farthest screw. The displacement cloud map illustrated that the peak displacement of fixation in the two groups was 1.91 mm and 1.26 mm, respectively, with the in situ group exhibiting less displacement than the reduction group (Fig. 12).

## Discussion

So far, there are no clear guidelines for correcting intercalation deformities for valgus-intercalated femoral neck fractures [26]. This type of femoral neck fracture has traditionally been considered stable in clinical settings [27, 28]. However, it is, in fact, partly unstable, exhibiting valgus and retroversion deformity of the femoral head, making it susceptible to secondary fracture displacement and internal fixation failure. The fracture is often associated with varying degrees of abduction and retroversion of the femoral head, leading many scholars to advocate for the correction of these deformities. Reduction can restore normal anatomy and trabecular weight-bearing lines, helping avoid unnecessary bone remodeling. It is conducive to the recovery of limb length and function and facilitates the reopening of retinaculum vessels after reduction, thereby reducing complications such as osteonecrosis of the femoral head.

In this study, finite element analysis revealed that the pressure on the femoral head and the fracture end in the reduction group was less than that in the valgus-intercalated group. Notably, the peak stress of the normal femur and abduction-embedded femur under a 1400N load was concentrated near the fracture line, but the stress peak value in the reduction group was lower than that in the valgus-intercalated group. This aligns with the research findings of Dai et al. [29]. Simultaneously, under the same load, the peak stress of the femur in the reduction group was lower than that in the valgus-intercalated group, with stress in the reduction group concentrating on the end of the femoral head and the medial side of the femur. In contrast, the peak stress in the valgus-intercalated group was concentrated near the fracture line. This suggests that the reduced femur exhibits a better and more scientifically sound mechanical model. Additionally, in the valgus-intercalated group, stress at the lateral fracture end of the femoral head was primarily concentrated in the lower area, making the femoral head more susceptible to torsion and varus under the influence of higher shear force. On the other hand, stress distribution at the fracture end section of the reduction group was more uniform and dispersed, effectively reducing the trend of stress concentration below. This further indicates that the stress dispersion effect of the reduction group is stronger than that of the valgus-intercalated group. To some extent, better stress dispersion can help prevent femoral head amputation and hip varus. Regarding the overall stress of the femur, the peak stress in the reduction group was lower than that in the valgus-intercalated group. Observing the stress model of the proximal femur in the reduction group revealed that the overall stress in the proximal femur in the reduction group was less than that in the valgus-intercalated group. This not only indicates smaller stress on the femur, promoting the healing of the fracture end, but also suggests that its mechanical structure is more scientifically designed, reducing the risk of internal fixation failure.

In terms of internal fixation and femoral displacement, the peak displacement in both groups was primarily concentrated at the top and gradually decreased downward, affirming the effectiveness of the model. However, the peak value of internal fixation and femoral displacement in the valgus-intercalation group was less than that in the reduction group. This disparity suggests that the in situ configuration can effectively reduce the risk of fracture end displacement and hip varus, as displacement serves as a reflection of fracture fixation to a certain extent.

Valgus femoral neck fractures are typically treated with internal fixation, especially using parallel cannulated screws in situ [30, 31]. Nevertheless, related studies have reported secondary operation rates of 10%-20%, nonunion rates of 10%, and femoral head necrosis rates of approximately 20% [32, 33]. The Femoral Neck System (FNS), introduced by DePuy Synthes in 2017, is a novel internal fixation system characterized by minimal trauma and a straightforward procedure [34]. It combines the advantages of internal fixation with hollow screws and enhances femoral neck preservation, promoting bone healing. Research has demonstrated [35] that peripheral nail fixation with ICS can compromise the residual blood supply of the femoral neck, while central nail implantation with FNS significantly reduces damage to the residual blood supply, thereby lowering the incidence of nonunion and osteonecrosis of the femoral head—an essential consideration for prognosis. Despite this, there is ongoing controversy regarding the indications and outcomes of these two surgical methods. Our findings indicate that the peak stress of internal fixation in the reduction and valgus-intercalation groups is concentrated in the fracture line area. Moreover, the peak stress of ICS internal fixation in both groups is lower than that of FNS, suggesting that ICS effectively conducts stress during post-reduction fixation, reduces stress in the fracture area, and exhibits superior stress dispersion and shear resistance compared to FNS.

This study has several limitations: (1) To prevent stress singularity, the femur's surface was smoothed, potentially differing slightly from mechanical experiments and clinical results. (2) Muscle effects on force were removed for more intuitive observation of results. (3) Before examination, bone and internal fixation material properties were simplified to isotropic composition, deviating from the bone's inherent material property distribution. (4) The study employed a single vertical loading method, while the actual hip joint is surrounded by muscle attachments with a more complex anatomical structure and force distribution. (5) This experiment serves as a preliminary basic study, utilizing only finite element analysis, with cadaver biomechanical experiments yet to be conducted. The research group plans to pursue further biomechanical experiments in subsequent investigations.

## Conclusion

In conclusion, our finite element analysis investigated the biomechanical impact of in situ or post-reduction fixation in valgus-intercalated femoral neck fractures. Additionally, we compared the biomechanical characteristics of ICS and FNS internal fixation in these scenarios. The findings demonstrated that stress levels in the femur, internal fixation device, and femoral fracture end were significantly lower after reduction compared to the in situ group. Conversely, the in situ group exhibited lower displacement in both the femur and the internal fixation device. Regardless of the reduction or in situ group, ICS consistently demonstrated lower stress and displacement compared to FNS.

## Data Availability

Follow-up regarding the perioperative use of hemostatic agents for intertrochanteric fractures in older femoral patients is not complete, so the dataset analyzed in this study is not publicly available but is available to the corresponding author on reasonable request.

## Abbreviations

FEMs: Finite element models
CS: Cannulated screw
FNS: Femoral neck system
ICS: Inverted cannulated screw
DHS: Dynamic hip screw
FEA: Finite element analysis
3d: Three dimensions
